# Neurodevelopment Outcome in Children with Fetal Growth Restriction at Six Years of Age: A Retrospective Cohort Study

**DOI:** 10.3390/ijerph191711043

**Published:** 2022-09-03

**Authors:** María José Benítez Marín, Juan Antonio Blanco Elena, Jesús Marín Clavijo, Jesús Jiménez López, Daniel María Lubián López, Ernesto González Mesa

**Affiliations:** 1 Medicine School, Malaga University, 29071 Málaga, Spain; 2 Obstetrics and Gynecology Service, Virgen de la Victoria University Hospital, 29010 Málaga, Spain; 3 General Surgery Service, Infanta Margarita Hospital, 14940 Córdoba, Spain; 4 Arts and Architecture Department, Málaga University, 29071 Málaga, Spain; 5 Obstetrics and Gynecology Service, Regional University Hospital of Malaga, 29011 Málaga, Spain; 6 Surgical Specialties, Biochemistry and Immunology Department, Málaga University, 29071 Málaga, Spain; 7 Biomedical Research Institute of Malaga (IBIMA) Research Group in Maternal-Fetal Medicine, Epigenetics, Women’s Diseases and Reproductive Health, 29071 Málaga, Spain; 8 Department of Obstetrics and Gynecology, Faculty of Medicine, University Hospital of Jerez de la Frontera, University of Cadiz, 11407 Cadiz, Spain

**Keywords:** fetal growth restriction, neurodevelopment, cognition, motor development, communicative development, development delay, brain sparing

## Abstract

Objective: This study aimed to describe neurodevelopment in fetal growth restriction children at the age of six. Secondly, we tried to demonstrate influencing factors that can improve or exacerbate this development, as well as predictive factors that might select a population at risk to assist with early childhood support. Method: It was a study of 70 children affected with FGR. FGR was based on these definitions: birth weight below the 3rd percentile or birth weight below the 10th percentile with an abnormal hemodynamic Doppler study. Neurodevelopment was assessed at 6 years old by means of Batelle Development Inventory. A global development quotient under a 100 score was considered a neurodevelopment delay. All variables regarding pregnancy care, delivery episode, postpartum, neonatal care, sociodemographic issues, and the need for support in the first years were studied. Results: The mean gestational age at diagnosis was 33.14 weeks (standard deviation (SD = 4.31), with 32.9% of early-onset diagnoses. The mean gestational age at delivery was 35.61 (SD = 3.21), and the cesarean rate was 64.3%. The average age of the children at the moment of the evaluation was 76.20-month-old (SD = 3.70). The mean global development quotient was 97.28 (SD = 13.97). We were able to record a 57.1% of global development delay. In the cases of cognition, only 17.1% of the children registered a delay. Motor and communication skills were the most frequently affected. We discovered that socioeconomic status was positively related to the global development quotient, as well as both gestational age at delivery and middle cerebral artery pulsatility index was positively related to the global development quotient. Conclusions: We found a higher neurodevelopment delay rate (57.1%). We could relate a higher gestational age at delivery and a higher MCA percentile with better global neurodevelopment quotients.

## 1. Introduction

Fetal growth restriction (FGR) is a pregnancy complication that occurs in approximately 10% of pregnancies worldwide. In this complication, fetuses cannot reach their predestinated genetic weight. Its multiple causes could be divided into two groups: due to placental insufficiency supply and non-placental origin (genetics or chromosomal disorders, congenital infections, or metabolic disorders). The classical definition of FGR was a fetus whose estimated weight was below the 10th percentile. Doppler study incorporation allows for differentiation between genetic small for gestational age fetuses and real FGR due to insufficiency of placental supply [1,2].

FGR could be divided into two groups depending on its onset, placental ischemic degree, and chronicity: early-onset and late-onset. Early-onset FGR is associated with early and severe ischemic placental involvement. It is associated with a high uterine artery pulsatility index measurement [3] and an elevated rate of early-onset preeclampsia, close to 70% [4]. Usually, umbilical artery (UA) Doppler is pathological at diagnosis, and associated with elevated severe neonatal outcomes [5]. Induced prematurity is a frequent outcome due to fetal deterioration, with high morbidity and mortality rates. Late-onset FGR has the same etiopathogenic basis, although it is a minor, late, and acute condition [6]. Its association with preeclampsia is less frequent, close to 8–15% [4,7]. UA usually is normal at diagnosis, even if they have circulation redistribution as a middle cerebral artery (MCA) or the ratio measurement is abnormal [7,8]. Commonly, neonatal morbidity rates are lower, but stillbirth and mortality are higher [7].

Adverse neonatal outcome in FGR has been exhaustively researched. TRUFFLE study described short-term outcomes in a cohort of fetuses with FGR. Prematurity was strongly associated with adverse neonatal outcomes, being more frequent neonatal sepsis and bronchopulmonary dysplasia. Adverse outcomes were more frequent at a lower gestational age at delivery or associated with hypertensive states [2].

Childhood and adulthood development could be affected by FGR [9]. Recently, interest in long-term outcomes is increasing. Specifically, cognitive and motor development, as well as brain structure development, is a primary field of study. The fetal period and early childhood are sensitive stages where genomic interactions with the environment occur as organs and systems acquire their long-term functions. Induced prematurity is a risk factor for adverse neurological outcomes. Those children could be affected by visual and auditory disabilities, coordination disorders, cognitive, and behavioral conditions and, in extreme cases, cerebral palsy [10]. Adverse neurological outcomes could be due to prematurity or as a consequence of FGR, although this connection is complex to link. In both cases, motor, cognitive and behavioral development could be affected by a hostile environment.

Multiple studies in animal models have demonstrated changes in brain structure. More precisely, induced hypoxia in animals has shown a reduction in neuron quantity [11] and a significant modification of dendritic arbors [12]. Other studies have demonstrated a delay in myelin production that could affect normal nervous conduction in the early stages [13,14]. This process is crucial to cognitive development in childhood. Furthermore, those changes have been appreciated in different concentrations of metabolites and neurotransmitters [15,16,17]. Studies in human models have also demonstrated a decrease in brain volume [18,19,20], a reduction in grey matter volume [18,21,22], as well as differences in white matter [23,24] and gyrification patterns [25] in FGR children. These modifications could affect cognitive, motor, and behavior development.

Poor cognitive development, behavioral disabilities, and academic difficulties have been related to FGR. Different studies have shown a higher incidence of neurodevelopmental disabilities in premature infants with FGR compared with preterm infants with adequate growth [26,27,28,29]. Late-onset FGR has also been related to cognitive disabilities and academic difficulties, but published data are contradictory and differences between groups tend to be minimal [30,31,32,33,34].

Even though the relationship between Doppler alterations and neonatal outcomes has been well established, the relationship with neurodevelopment has not been yet elucidated. Different studies have tried to link umbilical and middle cerebral artery alterations to cognitive and motor disabilities in childhood [35,36,37]. Classically, brain sparing has been associated with adaptative phenomena, but more recent studies have associated this process with poor cognitive and behavioral development [38,39,40,41,42,43,44]. In a recent systematic review, we were able to connect poor intelligence quotient results in children with brain sparing, but an association with motor or behavioral disabilities was difficult to link. These could be due to heterogeneity in the studies analyzed, great variety in both specific tests and ages of assessment, different definitions of brain sparing and lack of control of confounders [45]. In severe cases, the deleterious consequences of brain sparing on neurodevelopment could overpass the benefits of the sparing, leading to a wide spectrum of clinical manifestations [45].

We believe that FGR causes development disabilities in adaptative, motor, communication, and cognitive spheres during childhood. This study aimed to describe neurodevelopment in FGR children at six years of age. Secondly, we tried to demonstrate influencing factors that can improve or exacerbate this development, as well as predictive factors that might help us select a population at risk to assist with early childhood support.

## 2. Materials and Methods

### 2.1. Design and Population

This study was designed as a retrospective cohort study, in which we selected a group of children with fetal growth restriction born in 2015 at University Hospital Carlos Haya, Málaga, Spain. Inclusion criteria were based on FGR definition: less than the 3rd percentile birth weight or less than the 10th percentile birth weight with abnormal hemodynamic Doppler study. The abnormal hemodynamic study was defined as a pulsatility index (PI) of umbilical artery (UA) above the 95th percethe ntile, PI of middle cerebral artery (MCA) below the 5th percentile, cerebroplacental ratio (CPR) below the 5th percentile, or PI of uterine arteries above the 95th percentile. The CPR was calculated by dividing the PI of the MCA by the PI of the UA. A CPR below the 5th percentile was defined as brain sparing effect. Exclusion criteria were structural and chromosomal abnormalities, multiple pregnancies and small for gestational age. Once approval was obtained from the regional ethics committee, recruitment started in 2021. Parental consent was obtained before starting the procedure. Data were collected from clinical records, parents’ reports, and individually assessed children with Battelle Developmental Inventory (screening test).

### 2.2. Neurodevelopment Follow-Up at 6 Years Old

At the age of six, a Batelle Developmental Inventory (BDI) screening test (Spanish Edition) was performed prospectively [46]. We selected this screening test because it has a good correlation with the total inventory. The correlation level was 0.96 on all scales, except for the cognitive scale which was 0.92 [47]. This battery includes 96 items divided into five scales: personal-social, adaptive, motor, communicative and cognitive scale. Finally, a global score was obtained and converted into an equivalent developmental age on each scale. The items are presented in a standardized format, specifying the behavior or characteristic to be evaluated. This evaluation was performed individually and the average time to complete the test was approximately one hour. Information was obtained by means of direct observation, parental interviews, and direct children assessment. The global development quotient was obtained using Moraleda’s formula as follows: dividing the equivalent developmental age by the real age multiplied by 100 [47]. We considered the upper limit of the range at the final equivalent age of the BDI to calculate this ratio. Children were considered to have a developmental delay if their score was lower than 100 [48].

### 2.3. Parent Reports

At the same time that the children were assessed, a questionary was provided to the parents. In this report, they were requested to provide information about sociodemographic items and children’s issues during the first years of life (necessity of early child support, academic difficulties, and illnesses during childhood). Completion of the questionary required circa 15 min.

### 2.4. Data Collection

Medical records were searched for data about pregnancy and neonatal care during the first days of life. We registered variables about pregnancy care, delivery episode, postpartum, as well as weight, length, and head circumference of the neonate. We recorded the days of neonatal intensive care unit (NICU) admission and adverse neonatal outcomes if this was the case. NICU admission was considered when newborns required invasive care or close monitoring by neonatal pediatricians. Those children who remained with their mothers in the maternity ward were not considered in this group.

### 2.5. Statistical Analysis

Firstly, we carried out a descriptive analysis to detail the frequency distribution of the different variables in the cohort, as well as the frequency distribution of the developmental delay on each scale and global scale. Secondly, we explored the association between global development quotient and sociodemographic, pregnancy, Doppler measurement, delivery, and neonatal variables using the Student’s *t*-test or ANOVA test analysis.

Thirdly, multivariate analysis was accomplished to analyze moderating factors. Linear regression analyses were performed to examine the effect of (1) sociodemographic factors, (2) Doppler measurements, birth weight and age at delivery, and (3) postnatal factors. We incorporated all sociodemographic factors (maternal and paternal studies level, socioeconomic status, maternal and paternal occupational status, and separated parent status) in the first model. In the second model we included doppler measurements of UA, MCA, and CPR, birth weight and gestational age at delivery. In the third model, we included gestational age at delivery, gender, adverse neonatal outcomes, early childcare, academic difficulties, and nursery assistance. For all analyses, a p-value below 0.05 was considered significant. Data were processed and analyzed using the Statistical Package for the Social Sciences (SPSS), Version 22.0 (SPSS Inc., Chicago, IL, USA).

## 3. Results

### 3.1. Population

**Figure 1:**
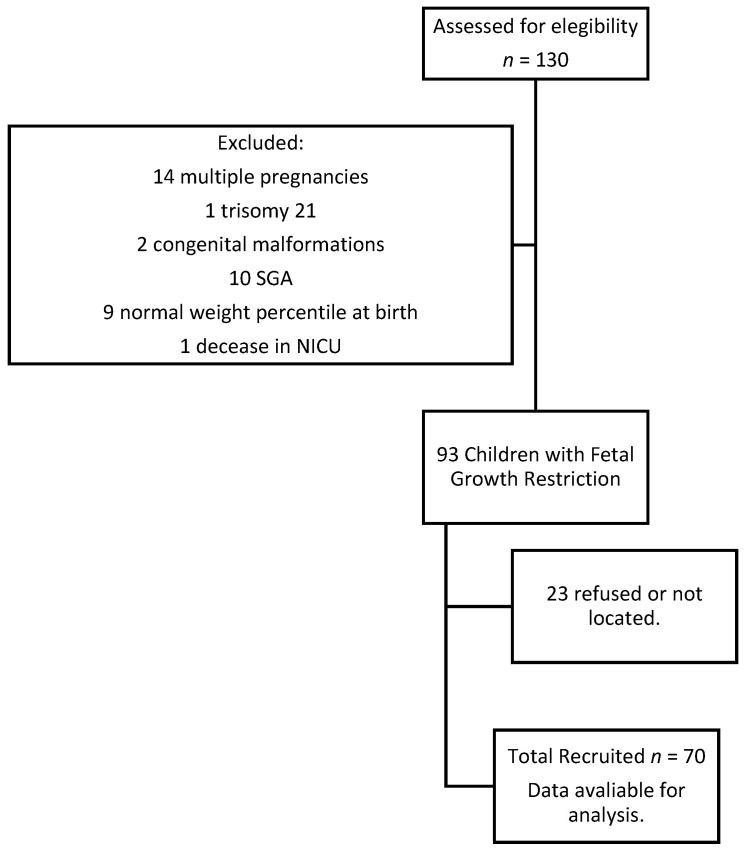
Flow-diagram: Participants in the study.

Initially, 130 patients with growth difficulties during pregnancy and born in 2015 were located in our documentary system. After applying exclusion criteria, a total of 37 children were excluded (14 multiple pregnancies, one trisomy 21, 2 congenital malformations, 10 small for gestational age, 9 children with a birth weight above the 10th percentile, and one newborn deceased during his NICU admission). Of the remaining 93 children, 70 parents agreed to carry out the neuropsychological assessment and consented to the revision of the medical records (Figure 1). Children were found to be between 70–84 months old during the evaluation. The remaining 23 children could not be assessed and BID was unavailable. Recruitment was laborious because some parents could not be located and some refused the interview due to lack of time or interest. All the cases of FGR diagnosed in our hospital that met the inclusion criteria were considered for the aim of this study. Being a single-center study no case sampling was performed. All eligible cases were thus included. Finally, we recruited seventy 6-year-old children. The group was representative of the initial population with a confidence level of 95%, a type II error of 0.6, and a statistical power of 94%.

### 3.2. Characteristics of Population Participants

Table 1 summarizes sociodemographic and delivery characteristics. The most frequent maternal and paternal educational level was secondary degree (54.3% and 51.4%). One mother and father declared no studies, but we did not reference them in the table. The most frequent socioeconomic status was medium (77.1%) and most parents were active workers. The mean gestational age at diagnosis was 33.14 weeks (SD = 4.31), with 32.9% of early-onset diagnoses. The mean fetal weight at diagnosis was 1616.38 g (SD = 660.25). The preeclampsia rate was 34.3%. In early-onset FGR, the preeclampsia rate was 60.9%, while in late-onset FGR was 21.7%. AU Doppler was registered in 65 cases, of which 20% were above the 95th percentile. In the case of MCA, only 56 were recorded and 41.4% were below the 5th percentile. CPR was calculated in 61 children, with pathological results in 50%.

The mean gestational age at delivery was 35.61 (SD = 3.21) and the cesarean rate was 64.3%. Arterial blood cord pH only was registered in cesarean section deliveries and the mean was 7.27 (SD = 0.09). In 23.5% of cases, this determination was less than 7.24. The average birth weight was 1848.30 g (SD = 589.74). The mean NICU admission was 15.57 days (2–127 days). Forty-one-point-four percent of the children did not require intensive care. Table 2 summarizes adverse neonatal outcomes, being neonatal respiratory distress syndrome and neonatal sepsis were the most frequently registered. No cases of intraventricular hemorrhage, periventricular leukomalacia, or other neurological complications were recorded.

### 3.3. Neurodevelopmental Outcome at 6 Years of Age

The average age of the children at the moment of the evaluation was 76.20-month-old (SD = 3.70). Non-major health problems or neurosensory disabilities were reported by parents, except for a child with bronchopulmonary dysplasia. Two cases of conditions encompassed within autism spectrum disorder (ASD) were disclosed. One of these children had difficulties regarding adaptative skills, and the other had poor global development. Thirty-six-point-eight percent of children needed early child support, during an average period of 3.39 years. Sixteen-point-seven percent of the children are currently following this stimulation program. Parents reported that 27.1% of children with learning disabilities. 

Table 3 and Table 4 summarizes the mean quotient and the percentages of children with developmental delay in the different scales. The mean global development quotient was 97.28 (SD = 13.97). We were able to record a 57.1% of global development delay. In the cases of cognition, only 17.1% of the children registered a delay. Motor and communication skills were the most frequently affected.

### 3.4. Bivariant Analyses

Table 5 summarizes the mean value and standard deviation for the global development quotient for each variable studied. We were able to find a higher quotient in children whose mothers had completed secondary or university studies (*p* < 0.05), as well as in the case of active employment status (*p* < 0.05). Similar results were accounted regarding the father study level and employment status but this finding did not reach the level of statistical significance. Socioeconomic status was also significantly associated with the mean of the quotient, being the case that which middle and high levels reach better quotients (*p* < 0.05).

Early-onset FGR were linked to a lower quotient than late-onset (91.51 versus 99.92), reaching this data statistical significance (*p* < 0.05). When UA Doppler was studied, we found a significantly lower quotient in the cases with pathological measurement (98.30 in normal measurement versus 89.90 in pathological measurement, *p* < 0.05). These results were similar in the case of pathological MCA or CPR, but this did not reach statistical significance. We found a better quotient in the case of the feminine gender (*p* < 0.05).

Table 6 summarizes the mean and standard deviation for each variable in the subgroup of neurodevelopment delay. Lower gestational age and fetal weight at diagnosis were observed in the case of neurodevelopment delay (*p* < 0.001). In the same way, we found a higher UA measurement and lower MCA and CPR measurements in the neurodevelopment delay group (*p* < 0.05). At the moment of delivery, we found a lower mean gestational age, birth weight, length, and head circumference in the neurodevelopmental delay group with statistical significance (*p* < 0.001). We could not find a difference in the quotient regarding the type of delivery, arterial/venous blood cord pH, or cardiotocographic register. Although the children that received breastfeeding obtained a better quotient value, this result did not reach statistical significance.

Among the neurodevelopmental delay group, children required more days of NICU care. Table 7 summarizes the mean quotient in case of adverse neonatal outcomes. Children that underwent neonatal respiratory distress syndrome (NRDS), neonatal sepsis, or bronchopulmonary dysplasia (BPD) obtained significantly lower quotients (*p* < 0.05). The worst outcomes were related to children needing intubation or developed BPD. Children with other conditions achieved lower quotient values, but this result was not significant. We should notice that these were pathologies with a low incidence among the studied subject.

Table 8 summarizes the results obtained when children required early child support or had any learning disability. The poorest outcomes were accounted for in case they needed any support therapy or had academic difficulties, reaching statistical significance in case of early child support, physiotherapy support, or learning disabilities (*p* < 0.001). Better results were achieved when nursery school was attended (no significance).

### 3.5. Multivariate Analyses

A linear regression analysis was performed to control the mediator factors. In the first model sociodemographic factors were included. We detected that socioeconomic status was positively related to global development quotient (*F*(1,62) = 9.41, *p* = 0.003). The R^2^ value was 0.132, showing that 13% of the effect is explained by differences in socioeconomic status. 

The second model included Doppler variables, birthweight, and gestational age at delivery as parameters. We could find that both gestational ages at delivery and MCA pulsatility index were positively related to the global development quotient (*F*(2,56) = 8.45, *p* = 0.001). The R^2^ value was 0.232, so both variables could explain the 23% of the effect.

In the third model, we included postnatal variables. We were able to find that both learning disabilities and the need for early child support were negatively related to the global development quotient (*F*(2,58) = 15.33, *p =* 0.0001). These findings could be explained by higher rates of severe FGR and extreme prematurity among these children. These features make them more prone to receive early stimulation support than children with non-severe FGR or non-extreme prematurity. In the same way, they are more susceptible to academic difficulties. Table 9 sums up these models.

## 4. Discussion

We performed a study to assess the neurodevelopment of children with FGR at 6 years of age. We have demonstrated higher neurodevelopment delay rates in these children. At the same time, we have proved a positive relationship between gestational age at delivery and MCA percentile with the global development quotient.

The relationship between hemodynamics disturbances, prematurity in FGR, and adverse neonatal outcomes has been well established. Assessing neurodevelopment in children with a history of FGR is more intricate. Neurodevelopment is a continuous process with different stages that can be influenced by multiple postnatal factors, both protective and risk factors. In the same way, the severity of hemodynamic alterations in FGR, as well as in prematurity, could involve a deleterious effect in the process. Our global developmental delay was 57.1%, higher than that found in other studies (close to 10–20%) [35,49,50]. This divergence could be due to different ages and methods of assessment. We evaluated the children at a late age (6 years old) by means of BDI, which assesses completely all neurodevelopment areas.

Baschat et al. (2009) found an increased risk of global retardation, cerebral palsy, and neurosensory abnormalities among FGR children with the reverse flow of the UA. They also determined that birthweight, gestational age at delivery, and neonatal adverse outcomes were strong predictors of adverse neurodevelopment [35]. In our bivariate analysis, similar results were found. A significantly lower gestational age and birth weight were found in children with developmental delays. Length of stay in the NICU was longer in children with developmental delays. We only could find a relationship between NRDS, neonatal sepsis, and BPD with worse scores in the global development coefficient. However, we could not associate perinatal outcomes with the global neurodevelopment coefficient in the multivariate analysis. This finding could be due to the low perinatal morbidity rates recorded in our study cohort.

We found that prematurity and brain sparing could be risk factors for impaired neurodevelopment. We saw a statistical association between global development quotient and gestational age at delivery and MCA pulsatility index percentile. Previous studies have related prematurity with poor neurodevelopment. In the GRIT study, the investigators evaluated the possibility of immediate or late delivery, always under safety conditions for the fetus. They found similar mortality rates in both groups. When they assessed neurodevelopment at 2 years of age, they observed comparable rates of neurodevelopment disabilities. However, when they evaluated extreme prematurity (24–30 weeks) they encountered higher rates of neurodevelopment disabilities in the immediate delivery group than in the late delivery one (13% versus 5%), as well as a poorer development quotient. A tendency to reduce morbidity and mortality in late delivery was noted [51]. When they assessed children at 6–13 years of age, they could not find differences in motor, cognitive development, or behavioral disturbances [52].

Therefore, in early-onset FGR, prematurity plays a role in neurodevelopment, mainly in psychomotor development, independently of the severity of the FGR and hemodynamic disturbances [49]. The impact on psychomotor development is more important in extreme prematurity (before 28–29 weeks of gestation) [50,53]. Besides cerebral palsy rate is between 4–18% before 32 weeks of gestational age, being higher in early prematurity [35,53]. In the same manner, Guellec et al. (2011) found a higher impact on cognitive development in FGR fetuses born before 28 weeks (37.5%) than in older fetuses. However, the result was similar to fetuses with adequate weight born before 28 weeks of gestation (38.2%). This result was non-significant [53]. Our percentage of cognitive delay was 17.1% across the entire cohort. For us, psychomotor and cognitive consequences are difficult to demonstrate because our prematurity rate before the 28th week of gestation was 2.9%.

Contrary, in late-onset FGR, gestational age is not a determinant for neurodevelopment. The DIGITAT study found that birthweight below the 2.3rd percentile is the strongest predictor for abnormal neurodevelopment in fetuses born between 36–41 weeks. They conclude that expectant management could deteriorate birthweight and neurodevelopment at 2 years of age [54].

The brain sparing effect is more controversial. Classically, brain sparing has been defined as a protective phenomenon by means of which the brain obtains nutrients and oxygen for the maintenance of its proper function. Recent studies have demonstrated the contrary. Brain sparing is a risk factor for brain development, specifically for adaptative, motor, communicative and cognitive development. Brain sparing has been related to smaller head circumferences [43] and smaller brain volume at delivery [55,56]. We could not find this relationship in our cohort, in which head circumference was similar to non-brain-sparing FGR fetuses at delivery (data not shown).

Scherjon’s group study could not relate the umbilical-cerebral ratio (UCR) with cognitive disabilities at 12 months and three years of age. However, these children had higher hyperactivity disorder rates and fewer words in their vocabulary. When children were assessed at five years of age, they could find visual potentials suggesting worse maturation profile and slower responses, as well as poor cognitive development with lower scores in intelligence quotient [41,42,43]. They only could find differences in behavior at the age of 11 [44].

Other studies have shown cognitive disturbances in brain sparing group at early ages: lower scores in habituation, motor, social interaction, and attention areas at birth [57]; poorest cognitive development at 2 years old [55] and at 3 years of age [40]. Monteith et al. (2019) could demonstrate that FGR with brain sparing resulted in lower scores in motor development tests than FGR without this condition [40].

In our systematic review, published the last year, we could connect poor intelligence quotient results to brain sparing in children with FGR. The relationship between brain sparing and motor or behavioral disabilities was difficult to assess. A good reason for that lies in methodological differences as children were assessed at different ages, when disabilities might not have yet appeared or may have already improved. On the other hand, the lack of control of the confounder could affect the results [45]. Our findings in this cohort are consistent with this trend. We have found that the MCA pulsatility index is positively related to the global development quotient. 

Beukers et al. (2017) and Richter et al. (2020) could not find any association between brain sparing and cognitive development. They could not find differences in intelligence quotient either [58,59]. Specifically, the umbilico-cerebral ratio was not associated with adverse outcomes, and birth weight and sociodemographic variables seemed to take a more important role [58]. Although we reached a relationship between MCA and the global neurodevelopment quotient, our cognitive delay rate was low (17.1%).

We noted a worse quotient in those children with an AU pulsatility index above the 95th percentile. However, we could not link the AU percentile to this quotient in the multivariate analysis. This could be due to fetuses with AU pathological measurements usually being preterm, with significant deterioration. Prematurity could be more decisive for neurodevelopment than the measurement of the UA pulsatility index itself. Studies have shown controversy about this connection. Some studies determine that it is a good predictor of early neurological complications or cerebral palsy but not for adverse cognitive outcomes [37,60,61]. Other studies relate it to worse cognitive development and motor outcomes, as well as an increased rate of cerebral palsy, especially in cases where diastolic flow is absent or reversed [36,62]. We could not prove it. We only had 12 cases of absent end diastolic flow velocity in the umbilical artery. At the same time, non-cerebral palsy was found in our cohort. A larger sample size would be necessary to demonstrate an association between the measurement of UA PI and its characteristics and neurodevelopmental outcomes.

Despite the importance of fetal maturation, neurodevelopment is a complex and continuous process in which multiple factors could influence the progress. In our study, we have associated higher socioeconomic status with better global development quotients. Other studies identified a higher parental educational level [63,64] and socioeconomic status [58,65] as linked to better cognitive results. These findings could be due to higher implications and knowledge of the neurological stimulation process by parents.

On the other hand, we could not identify early stimulation in early child support as a protective factor in this group of children. Initially, we have recognized a negative association between early child support, academic disabilities, and global development quotient. These findings could be due to children attending early stimulation being a group with severe FGR and prematurity with worse results. But finally, early stimulation is a protective factor for this subgroup of children in a certain way. Other studies could identify early stimulation as a protective factor [64].

We could not relate breastfeeding as a protective factor or postpartum depression as a risk factor against other studies that succeeded to do so. Breastfeeding has been associated with better cognitive development in term and preterm infants, specifically when its use is prolonged. However, this effect is moderate when confounders are adjusted [66]. Nevertheless, breastfeeding improves neurological development, being the effect more powerful in low-birth-weight children [67] and children with lower cognitive test scores [68]. A systematic review has shown worse cognitive, language, and behavioral development in children with mothers affected by postpartum depression [69]. Postpartum depression is usually associated with worse caregiving, affecting thus neurodevelopment. Its implications for motor development are more controversial. We could not find any link between these variables and global development. Although children with breastfeeding or non-depression postpartum had better scores, this difference was minimal and non-significant. Further studies and larger sample sizes are necessary to demonstrate these associations.

## 5. Strengths and Limitations

Our study has some strengths. First, we assessed children at 6 years of age, when most of the motor, cognitive, and communication maturing processes are already well established. Therefore, we could accurately appraise the overall development of these children. Second, we assessed them using a complete test; the Batelle Developmental Inventory (screening test). This test allowed us to attain a global conception of the child’s development on all scales. It also allowed for considering children with disabilities, thus rendering them suitable to participate in our study. In addition, the test had a good correlation with its diagnostic modality.

Our main limitation was the sample size. Despite testing most children, a moderate number of parents were not reached or refused to participate. Although we found associations in multiple respects, we were unable to reach statistical significance. On the other hand, we could not assess a control group of children without growth restriction, to evaluate significant differences between them. Despite this, we could find a high percentage of global developmental delay. A larger sample size and a control group could be necessary to reach significance.

## 6. Conclusions

We found a high neurodevelopment delay rate (57.1%), specifically in motor and communicative skills. Cognitive skills have been preserved in most cases, with a low rate of delay (17.1%). Based on the results of our study, we can link gestational age at delivery and MCA percentile to the global development quotient, in the way that a higher gestational age at delivery and a higher MCA percentile is related to better quotients. Brain sparing could be a not fully protective phenomenon. In severe cases, it could surpass its protective effect becoming a harmful event for development. More studies are necessary to determine the percentile cut-off in which this effect stops being protective.

## Figures and Tables

**Table 1:** Sociodemographic and delivery characteristics.

Variables	n (%)	MD (SD)
Maternal educational level		
primary	7 (10%)	
secondary	38 (54.3%)	
university	23 (32.9%)	
Paternal educational level		
primary	18 (25.7%)	
secondary	36 (51.4%)	
university/super	13 (18.6%)	
Maternal employment status		
Active	43 (61.4%)	
Unemployed	25 (35.7%)	
Paternal employment status		
Active	58 (82.9%)	
Unemployed	8 (11.4%)	
Socioeconomic status		
Low	10 (14.3%)	
Middle	54 (77.1%)	
High	4 (5.7%)	
Separated parents	21 (30%)	
FGR history	7 (10.1%)	
Amniocentesis	8 (12.1%)	
Pathological uterus artery (second trimester)	24 (34.3%)	
Smoker in pregnancy	16 (22.9%)	
Postpartum depression	14 (20%)	
Preeclampsia	24 (34.3%)	
Gestational age at FGR Diagnosis (mean)		33.14 (4.31)
Early onset	23 (32.9%)	
Late onset	46 (65.7%)	
Fetal weight at diagnosis		1616.38 gr. (660.25)
UA PI Percentile (mean)		61.52 (27.94)
Pathologic	14 (20%)	
MCA PI Percentile (mean)		15.69 (22.68)
Pathologic	29 (41.4%)	
CPR percentile (mean)		14.18 (22.74)
Pathologic	35 (50%)	
Gestational age at delivery		35.61 (3.21)
<28 weeks	2 (2.9%)	
28–32 weeks	10 (14.3%)	
32–37 weeks	28 (40%)	
>37 weeks	30 (42.9%)	
Type of delivery		
Eutocic	21 (30%)	
Instrumental	4 (5.7%)	
Cesarean section	45 (64.3%)	
Pathological Nonstresant test	31 (44.3%)	
Arterial blood cord pH		7.27 (0.09)
Venous blood cord pH		7.30 (0.07)
Apgar 5 min		9.63 (0.80)
Gender (feminine)	37 (52.9%)	
Birthweight (gr)		1848.30 (589.74)
Length at delivery (cm)		43.25 (5.04)
Head circumference at delivery (cm)		30.26 (3.37)
Days NICU admission		17.57 (26.78)
Breastfeeding	52 (74.3%)	

**Table 2:** Adverse neonatal outcome frequencies.

Variables	n (%)
NRDS	22 (31.4)
Neonatal sepsis	14 (20)
ROP	6 (8.6)
BPD	4 (5.7)
GMH	3 (4.3)
PDA	6 (8.6)
NEC	3 (4.3)
Intestinal Perforation	2 (2.9)
Acute kidney failure	2 (2.9)

NRDS: neonatal respiratory distress syndrome, ROP: retinopathy of prematurity, BPD: bronchopulmonary dysplasia GMH: Germinal matrix hemorrhage, PDA: patent ductus arteriosus, NEC: necrotizing enterocolitis.

**Table 3:** Mean of Quotient reached in each development scale for children.

Area	MD	SD
Personal-social	105	21.44
Adaptative	102.14	21.18
Gross Motor skills	101.57	22.05
Fine Motor skills	96.21	18.23
Motor skills	96.45	17.82
Receptive communication skills	98.26	13.82
Expressive communication skills	104.84	19.06
Communication skills	98.25	16.79
Cognition	106.97	12.38
Global index	97.28	13.97

MD: mean values, SD: standard deviation, n/s: not significant.

**Table 4:** Percentage of children with developmental delay in each of the scales.

Area	Developmental Delay	Not Developmental Delay
Personal-social	32.9%	67.1%
Adaptative	37.1%	62.9%
Gross Motor skills	52.9%	47.1%
Fine Motor skills	62.9%	37.1%
Motor skills	55.7%	44.3%
Receptive communication skills	62.9%	37.1%
Expressive communication skills	34.3%	65.7%
Communication skills	55.7%	44.3%
Cognition	17.1%	82.9%
Global index	57.1%	42.9%

**Table 5:** Mean values and standard deviations of global development coefficient in each variable studied.

Variables	Options	MD	SD	Statistical Analysis
Maternal educational level	Primary	87.85	15.28	*F*(3,65) = 2.60, *p* = 0.05
	Secondary	96.48	13.16	
	University	102.33	12.31	
Paternal educational level	Primary	90.73	14.02	n/s
	Secondary	99.17	13.69	
	University	97.44	12.36	
Maternal employment status	Active	100.50	13.58	*t*(66) = -2.01, *p* = 0.048
	Unemployed	93.88	12.09	
Paternal employment status	Active	97.46	14.22	n/s
	Unemployed	93.70	9.16	
Socioeconomic status	Low	85.55	14.01	*F*(2,65) = 4.50, *p* = 0.015
	Middle	98.39	13.31	
	High	103.06	5.31	
Separated parents	No	98.88	12.31	n/s
	Yes	93.7	16.93	
FGR history	No	96.49	14.35	n/s
	Yes	105.36	7.47	
Amniocentesis	No	98.55	12.7	*t*(64) = 2.74, *p* = 0.008
	Yes	84.58	21.92	
Pathological uterus artery (second trimester)	No	101.29	11.51	n/s
	Yes	95.60	12.53	
Smoker in pregnancy	No	98.18	12.66	n/s
	Yes	94.23	17.85	
Postpartum depression	No	97.94	14.11	n/s
	Yes	94.64	13.55	
Preeclampsia	No	98.73	14.53	n/s
	Yes	94.50	12.66	
Diagnosis FGR	Early onset	91.51	12.63	*t*(67) = -2.43, *p* = 0.018
	Late onset	99.92	13.94	
UA PI Percentile	Normal	98.30	14.13	*t*(63) = 2.02, *p* = 0.047
	Pathological	89.90	12.05	
MCA PI Percentile	Normal	99.08	12.90	n/s
	Pathological	93.76	12.06	
CPR percentile	Normal	100.06	14.37	n/s
	Pathological	94.30	10.59	
Type of delivery	Eutocic	99.53	11.84	n/s
	Instrumental	100.23	4.68	
	Cesarean section	95.96	2.26	
Non stresant test	Normal	99.57	12.02	n/s
	Pathological	94.39	15.82	
Breastfeeding	No	94.12	17.64	n/s
	Yes	98.37	12.47	
Birthweight percentile	≤3	96.54	14.39	n/s
	>3	101.72	10.66	
Length percentile at delivery	≤3	90.72	17.59	*t*(64) = -2.4, *p* = 0.019
	>3	99.93	12.23	
Head circumference percentile at delivery	≤3	96.36	12.52	n/s
	>3	98.22	15.56	
Gender	Feminine	102.57	13.94	*t*(68) = 2.35, *p* = 0.022
	Masculine	93.41	18.54	

FGR: Fetal growth restriction, UA PI Umbilical Artery pulsatility index, MCA PI: Middle cerebral artery pulsatility index, CPR: cerebroplacental ratio, MD: mean values, SD: standard deviation, n/s: not significant.

**Table 6:** Mean values and standard deviations in neurodevelopment delay subgroups.

Variables	Neurodevelopment Delay	MD	SD	Statistical Analysis
Gestational age at diagnosis	Yes	31.66	4.27	*t*(67) = −3.62, *p* = 0.001
	No	35.18	3.51	
Fetal weight at diagnosis	Yes	1408.20	639.61	*t*(66) = −3.33, *p* = 0.001
	No	1913.79	578.69	
UA PI Percentile	Yes	68.82	29.47	*t*(63) = 2.7, *p* = 0.009
	No	50.58	21.68	
MCA PI Percentile	Yes	9.62	11.90	*t*(57) = −2.82, *p* = 0.007
	No	25.91	31.68	
CPR percentile	Yes	9.03	17.64	*t*(59) = −2.27, *p* = 0.027
	No	22.13	27.46	
Gestational age at delivery	Yes	34.54	3.33	*t*(68) = −3.46, *p* = 0.001
	No	37.04	2.43	
Arterial blood cord pH	Yes	7.27	0.10	n/s
	No	7.27	0.08	
Venous blood cord pH	Yes	7.30	0.08	n/s
	No	7.30	0.06	
Birthweight	Yes	1654.65	610.22	*t*(68) = −3.40, *p* = 0.001
	No	2106.50	453.92	
Length at delivery	Yes	41.91	5.28	*t*(64) = −2.46, *p* = 0.016
	No	44.87	4.28	
Head circumference at delivery	Yes	29.26	2.66	*t*(63) = −2.71, *p* = 0.009
	No	31.43	3.77	
Days NICU admission	Yes	25.33	31.36	*t*(68) = 2.94, *p* = 0.004
	No	7.23	13.78	

Neurodevelopmental delay was considered when the global developmental quotient was below 100. UA PI Umbilical Artery pulsatility index, MCA PI: Middle cerebral artery pulsatility index, CPR: cerebroplacental ratio, NICU: neonatal intensive care unit, MD: mean values, SD: standard deviation, n/s: not significant.

**Table 7:** Mean values and standard deviations of global development coefficient in each neonatal outcome studied.

Variable	Options	MD	SD	Statistical Analysis
NRDS	No	99.61	14.33	*F*(2,67) = 3.30, *p* = 0.043
	CPAP	94.14	9.67	
	Intubation	83.39	18.36	
Neonatal sepsis	No	98.97	13.76	*t*(68) = 2.08, *p* = 0.041
	Yes	90.49	13.12	
ROP	No	98.14	13.36	n/s
	Yes	88.07	18.19	
BPD	No	98.16	13.38	*t*(68) = 2.20, *p* = 0.031
	Yes	82.73	17.59	
GMH	No	97.56	13.46	n/s
	Yes	90.87	26.28	
PDA	No	97.94	13.50	n/s
	Yes	90.21	18.19	
NEC	No	97.74	13.59	n/s
	Yes	86.88	21.66	
Intestinal Perforation	No	97.61	13.89	n/s
	Yes	85.80	16.29	
Acute Kidney Failure	No	97.61	13.89	n/s
	Yes	85.80	16.29	

NRDS: neonatal respiratory distress syndrome, ROP: retinopathy of prematurity, BPD: bronchopulmonary dysplasia GMH: Germinal matrix hemorrhage, PDA: patent ductus arteriosus, NEC: necrotizing enterocolitis, MD: mean values, SD: standard deviation, n/s: not significant.

**Table 8:** Mean values and standard deviations of the global development coefficient in each childcare variable studied.

Variable	Options	MD	SD	Statistical Analysis
Early Child Support	No	102.40	10.11	*t*(66) = 4.20, *p* = 0.0001
	Yes	89.49	15.19	
Speech therapy	No	99.89	11.18	n/s
	Yes	96.97	8.82	
Physiotherapy	No	102.23	10.27	*t*(59)=3.17, *p* = 0.002
	Yes	93.16	9.91	
Nursery school	No	96.31	12.55	n/s
	Yes	97.48	14.34	
Learning disability	No	102.43	9.39	*t*(68) = 6.31, *p* = 0.0001
	Yes	83.45	15.04	

MD: mean values, SD: standard deviation, n/s: not significant.

**Table 9:** Best fit linear regression models of different variables.

Model	Variables	Β	Standard Error	*t*	95%CI		*p*	R^2^
					Lower	Upper		
1	Socioeconomic status	10.45	3.40	3.06	3.64	17.27	0.003	0.132
	(Constant)	87.83	3.46	25.37	80.91	94.75	0.0001	
2	Gestational age at delivery	1.41	0.47	2.98	0.46	2.36	0.004	0.232
	MCA PI Percentile	0.17	0.06	2.62	0.04	0.30	0.011	
	(Constant)	43.98	16.73	2.62	10.46	77.50	0.011	
3	Learning disability	−11.88	2.96	−4.00	−17.82	−5.94	0.0001	0.346
	Need for Early Child Support	−7.33	2.58	−2.83	−12.51	−2.16	0.006	
	(Constant)	104.05	1.43	72.72	10.19	106.92	0.0001	

Variables with significant value included in the different models: socioeconomic level (low, middle, high), gestational age at delivery, MCAPI percentile, learning disability (yes or no), early child support (yes or no). CI: confidence interval.

## Data Availability

Not applicable.
